# Community-engaged health outreach program contributes to health service, research, and education in rural areas

**DOI:** 10.3389/frhs.2026.1749125

**Published:** 2026-05-12

**Authors:** Zizipho Z. A. Mbulawa, Bomikazi Majeke, Bantubonke Ntamo, Pumla Kwake, Ernesto Rosales Gonzalez, Nomawethu Matanzima, Charles B. Businge, Busisiwe Cawe, Lindiwe M. Faye

**Affiliations:** 1 School of Pathology, Walter Sisulu University, Mthatha, South Africa; 2 National Health Laboratory Service, Nelson Mandela Academic Hospital, Mthatha, South Africa; 3 School of Public Health, Walter Sisulu University, Mthatha, South Africa; 4 Tabase Community Health Centre, Eastern Cape Department of Health, Mthatha, South Africa; 5 Department of Family Medicine and Rural Health, Walter Sisulu University, Mthatha, South Africa; 6 Department of Nursing, Walter Sisulu University, Mthatha, South Africa; 7 Department of Obstetrics and Gynaecology, Walter Sisulu University, Mthatha, South Africa

**Keywords:** community engagement, eastern cape, education, health, outreach, research

## Abstract

A community-engaged health outreach program is a temporary intervention designed to address health risks faced by a specific population. This report examines the impact of a community-engaged health outreach program conducted at the Tabase Clinic in Mthatha, Eastern Cape Province, South Africa. This tripartite health service, educational, and research outreach initiative was conceptualized and organized by Walter Sisulu University Faculty of Medicine and Health Sciences, National Health Laboratory Service, Eastern Cape Department of Health, and Tabase clinic committee. During the outreach, the Tabase clinic functioned as a one-stop multidisciplinary team polyclinic, comprising nurse-physician-paramedical teams from the Departments of Paediatrics, Urology, Surgery, Obstetrics and Gynaecology, Family Medicine, Physiotherapy, Pathology, Dentistry, Public Health, Social Work, Pharmacy, National Health Laboratory Service, Nursing, Orthotics and Prosthetics, and non-governmental organizations. There was a 6-fold increase in the number of clients who received healthcare at Tabase clinic during the outreach compared to regular days (from 50 to 60 to 388 clients). The attendees resided in villages that are up to 41 kilometers away from the Tabase clinic. The Tabase clinic catchment area has a high burden of hypertension, diabetes mellitus, epilepsy, pulmonary tuberculosis, extra-pulmonary tuberculosis (TB), asthma, schizophrenia, human immunodeficiency virus (HIV), cancer, cold, and flu. Males were the majority of attendees (53.1%) tested for HIV, a Men's Corner strategy attributed to this. Among those tested, 4.7% were newly diagnosed HIV cases, and clients were initiated on antiretroviral therapy. The health outreach also contributed to the learning of MBChB level four and six, honours, and masters’ students of the WSU Faculty of Medicine and Health Sciences. The major unmet need in the community was the lack of an ophthalmologist and an ear, nose, and throat specialist. All domains assessing perceptions of quality, relevance, and organization scored significantly above the neutral midpoint (Likert scale of 3; *p* < 0.05; R software, Version 4.3.1), confirming the strong acceptance of the program by the community. While logistics and educational delivery were not strongly scored. This outreach successfully bridged a vital gap in healthcare at the community level, enhancing community-based education and service.

## 1 Introduction

A community-engaged health outreach program is a collaborative approach that usually involves partnerships between academic institutions, research institutions, healthcare providers, and community members to improve health outcomes, enhance research relevance, and strengthen education (1–3). Although health outreach programs are temporary, they are an effective health intervention for reaching populations facing health risks (3). Community-engaged health outreach programs are necessary for rural areas and underserved populations, such as the majority of the Eastern Cape Province in South Africa (1). Community-engaged medicine and health science research activities are also limited in this region. The Eastern Cape Province of South Africa has experienced, and continues to experience, challenges in developing and implementing effective healthcare systems (4, 5). The Eastern Cape rural clinics lacked consistent access to essential medications and diagnostic services, contributing to poor chronic disease management and delayed care. Additionally, it has limited access to healthcare professionals and specialists (4–6). Despite these challenges, community-engaged health outreach initiatives have shown promise in bridging service gaps. Studies conducted in South Africa have demonstrated that mobile clinics and nurse-led outreach programs have significantly improved immunization coverage and antenatal care attendance (7, 8).

Medical schools are an essential source of healthcare professionals, community-informed research, services, and support for the communities in which they are located (9–11). Currently, there are two medical schools in the Eastern Cape Province: Walter Sisulu University (WSU) with its main campus located in Mthatha, and Nelson Mandela University (NMU) in Gqeberha. In response to the aforementioned challenges confronting the Eastern Cape Province, Walter Sisulu University's Faculty of Medicine and Health Sciences employs several strategic interventions, including community-engaged health outreach programmes. This report focuses on the Tabase Community Health Outreach Programme, outlining its contributions to health service delivery, education, and research.

Despite increasing recognition of the importance of health outreach in improving health outcomes, rural communities in the Eastern Cape province of South Africa continue to experience a disproportionate burden of communicable and non-communicable diseases, including HIV, tuberculosis, hypertension, diabetes, and cancer (12–14). These persistent challenges are driven by interconnected social determinants, including poverty, limited access to healthcare services, low health literacy, stigma, and structural barriers within the health system (15, 16). While community-engaged interventions are frequently implemented to address these gaps, there remains limited empirical and conceptual clarity on how such approaches systematically influence health behaviors, service utilization, and disease outcomes across different conditions (17). Furthermore, existing health strategies often operate in silos and inadequately integrate community-driven knowledge, resulting in missed opportunities for sustainable and contextually relevant health improvements. In addition, there is paucity of data on the organization and impact of multidisciplinary community health outreach in South Africa. Therefore, this study seeks to address this gap by examining how community engagement can be effectively leveraged to strengthen health literacy, improve care-seeking behaviors, and enhance disease prevention and management in rural Eastern Cape communities.

## 2 Context

The Tabase community-engaged health outreach was initiated by the Tabase clinic operational manager (Kwakhe Pumla) and WSU MBChB-3 community-based education and service tutor (Zizipho Mbulawa) during the community-based education activity when health service gaps, high sexually transmitted diseases burden and women health issues were identified in Tabase clinic catchment area. The idea was then shared with the Tabase clinic committee in which during the engagement they adopted the vision and requested that the Tabase health outreach must not only focus on the above mentioned area and should be an open health outreach to reduce stigmatization. They explicitly mentioned that it will be possible that the population we are targeting may be missed due to fear of stigma. After engaging with the Tabase clinic committee, the health outreach was shared with the Walter Sisulu University Faculty of Medicine and Health Sciences community engagement committee and with the Tabase community traditional/political leadership, both adopted the vision.

To organize the programme, there was continuous engagement, from the 17th of June to 26th August 2025 (2 h meeting, every second Tuesday) between members of Walter Sisulu University Faculty of Medicine and Health Sciences, National Health Laboratory Service (NHLS), Tabase clinic committee, and Eastern Cape Department of Health [OR Tambo district municipality, King Sabata Dalindyebo local municipality, Nelson Mandela Academic Hospital (NMAH), and Mthatha Regional Hospital]. The TB/HIV Care and Mthatha Women Support Centre were then later invited to join the Tabase health outreach programme.

**Figure 1:**
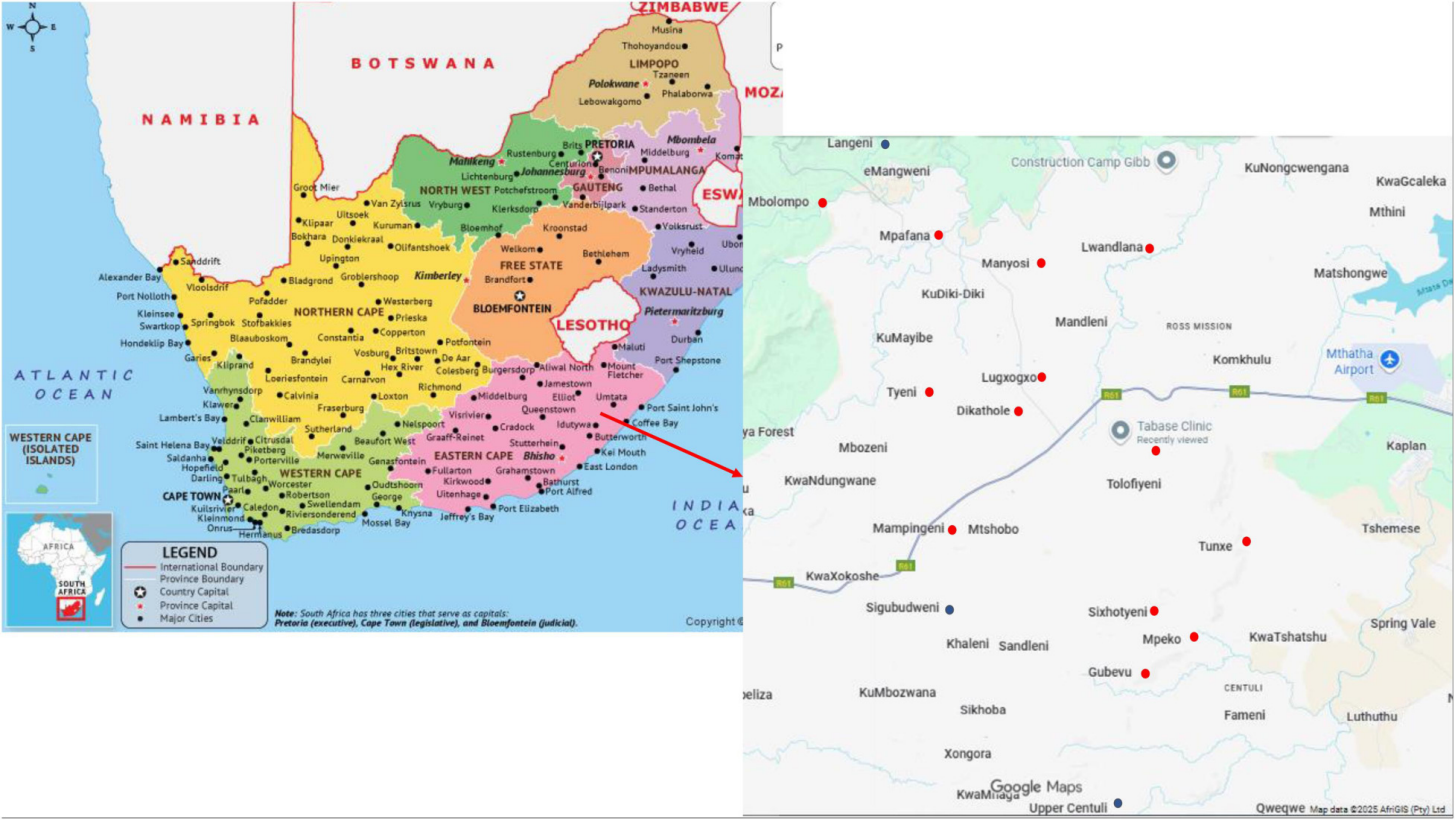
Map showing Africa, South African provinces, Eastern Cape Province, tabase clinic in tabase mission village, and surrounding catchment villages (18). The villages with round dots are where Tabase health outreach attendees reside. The red dots indicate some of the villages that are official Tabase clinic catchment villages. While the blue dots indicate villages that are not part of the Tabase clinic catchment area, but attended the outreach.

**Figure 2:**
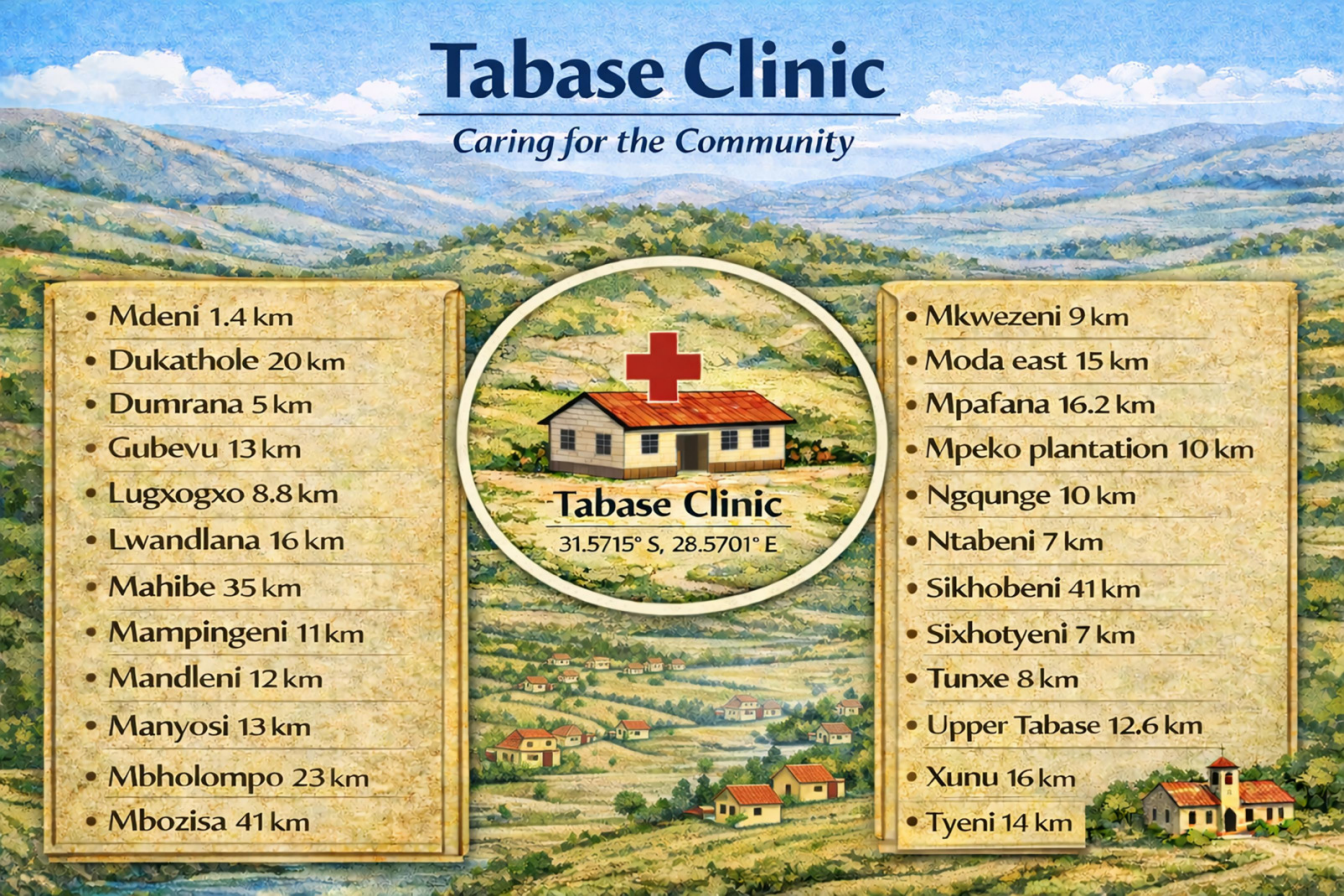
Spatial distribution of villages and distances to Tabase Clinic in a rural catchment area in the Eastern Cape Province, South Africa. The clinic, located at 31.5715°S and 28.5701°E, serves Tabase Mission and nearby communities. Distances from the clinic to each village are shown in kilometers, illustrating the geographic spread of the catchment area (Canva version 2026, Sydney, Australia).

The Tabase health outreach was held on the 28th of August 2025, at Tabase Clinic. The Tabase Clinic, located at geographical coordinates 31.5715° South and 28.5701° East, primarily serves the Tabase Mission and its surrounding communities (Figure 1). The catchment areas that are served by Tabase clinic include the following villages, Mdeni [1.4 kilometers (km) away from Tabase clinic], Dumrana (5 km), upper Tabase (12.6 km), Ngqunge (10 km); Sixhotyeni (7 km); Mahibe (35 km), Tyeni (14 km), Mbholompo (23 km), Manyosi (13 km), Mbozisa (41 km), Mampingeni (11 km), Dukathole (20 km), Lugxogxo (8.8 km), Lwandlana (16 km), Mandleni (12 km), Gubevu (13 km), Mkwezeni (9 km), Sikhobeni (41 km), Tunxe (8 km), Mpeko plantation (10 km), Ntabeni (7 km), Moda east (15 km), Mpafana (16.2 km) and Xunu (16 km, Figure 2). Tabase Clinic serves an estimated population of 17,578 individuals across all age groups. It is important to note, however, that the outreach activities were not confined to communities officially designated as part of the Tabase Clinic catchment area.

The Tabase health outreach was a one-day event that started at eight in the morning and ended at six in the evening. The program included educational talks on various health conditions, interactive sessions, distribution of educational materials, surveys on disease/infection knowledge, basic health checks, screenings, and consultations. The services provided included, but were not limited to, screening for tuberculosis (TB), human immunodeficiency virus (HIV), cervical, breast and prostate cancers, as well as the assessment and management of urological and surgical conditions, gynaecological services, oral and dental care, rehabilitation services (including physiotherapy and orthotics and prosthetics), maternal and child health services, mental health care, and the management of non-communicable diseases (Table 1).

**Table 1:** Institutions or organization's role in Tabase community health outreach.

Institutions/organizations participated	Partner's role and/services provided
WSU Faculty of Medicine and Health Sciences	Coordination, health education, basic health checks, screenings, consultations, and refreshments
National Health Laboratory Service	Coordination, health education, specimen collection, laboratory investigations, and refreshments
Eastern Cape Department of Health	Coordination, health education, basic health checks, screenings, and consultations
Tabase clinic committee	Coordination, community mobilization, and refreshments
Nelson Mandela Academic Hospital & Mthatha Regional Hospital	Coordination, health education, basic health checks, screenings, and consultations
TB/HIV Care	HIV testing and TB screening
Mthatha Women Support Centre	Counselling and psychosocial support

## 3 Key elements of the health outreach programme

### 3.1 Mobilization of the outreach teams

This tripartite health service, educational, and research outreach initiative was conceptualized and organized by Walter Sisulu University Faculty of Medicine and Health Sciences, NHLS, Eastern Cape Department of Health and Tabase clinic committee. The health professional workers from WSU Faculty of Medicine and Health Science, Eastern Cape Department of Health, TB/HIV care and Mthatha women support center were mobilized by invitation letters, posters, and word of mouth. This resulted in the establishment of a one-stop multidisciplinary team polyclinic, comprising nurse-physician-paramedical teams from the Departments of Paediatrics, Urology, Surgery, Obstetrics and Gynaecology, Family Medicine, Physiotherapy, Pathology, Dentistry, Public Health, Social Work, Pharmacy, the National Health Laboratory Service, Nursing, Orthotics and Prosthetics, and non-governmental organizations. In addition, the undergraduates (MBChB 4th year and MBChB 6th year) and postgraduate (Honours and Masters) students also participated (Table 2).

**Table 2:** Different health professionals, students and administrators who contributed in Tabase community-engaged health outreach.

Staff and students	Number of those provided service
Obstetrics and gynecologist	1
Women's health professional nurses	2
Pediatricians	2
Pediatrician professional nurse	1
Urologist	1
Breast surgeon specialist	1
Public Health Medicine specialist	3
Family medicine specialist	3
Orthotists and prosthetists	2
Physiotherapist	1
Dentists	2
Dental assistants	4
Professional nurses	10^a^
Pharmacist	1^b^
Staff nurse	2^a^
Community health care workers	8^a^
Lay counsellors	4^a^
Social worker	1
Phlebotomist	2
Medical scientist (Microbiologist & Virologist)	4
MBChB 4 & 6	16
Postgraduates in health sciences	18
Administrators	5^b^

a Include the clinic staff.

b Only clinic staff.

### 3.2 Mobilization of the community

This project was grounded in a high-impact community-engaged approach informed by principles of Community-Based Participatory Research (CBPR), Boyer's Scholarship of Engagement, and Ubuntu-informed relational ethics. Prior to implementation, both structured and informal consultations were conducted to co-identify health priorities and contextual barriers, ensuring that the intervention was responsive to locally articulated needs rather than externally imposed agendas. Tabase clinic committee representing the community members was actively involved in the co-design of the programme, contributing to decisions on outreach strategies, selection of outreach venue, timing of activities, and the range of services provided. The Tabase clinic was chosen over the community hall and school for its superior infrastructure. By using a venue with existing consultation rooms and a pharmacy, the team avoided the logistical challenge of transporting equipment to an off-site location.

The Tabase clinic committee and clinic staff further played a central role in community mobilization, facilitation of engagement activities, and the contextual adaptation of health messages to ensure cultural relevance and acceptability. This approach reflects a deliberate shift from traditional top-down service delivery towards a co-production model of knowledge and practice, in which community members are recognized as equal partners in both research and implementation. Grounded in Ubuntu principles of mutual respect, reciprocity, and collective responsibility, the project fostered trust, strengthened community–institution relationships, and enhanced the legitimacy and sustainability of the intervention.

Community mobilization for the outreach was led collaboratively by Tabase Clinic staff, clinic committee members, community leaders, and the outreach team. Multiple locally appropriate communication channels were utilized, including posters displayed at the clinic and community shops, as well as word-of-mouth dissemination through community networks. In addition, the outreach activities were broadcast via Unitra Community Radio (FM), a widely accessible platform within the O.R. Tambo District Municipality and King Sabata Dalindyebo Local Municipality, thereby maximizing reach and community participation.

### 3.3 Organization of the services during the outreach

The organizing team designated an area that focused on males, and it was called “Men's Corner” at the outreach. This was done in order to create a safe space, increase health-seeking behavior, and encourage engagement among men (19). The urologist, male surgeon, and one male nurse were tasked to focus more on the “Men Corner”; however, they were not limited to it. After registration, males were allowed to join the “Men's Corner” or continue in the normal queue.

**Figure 3:**
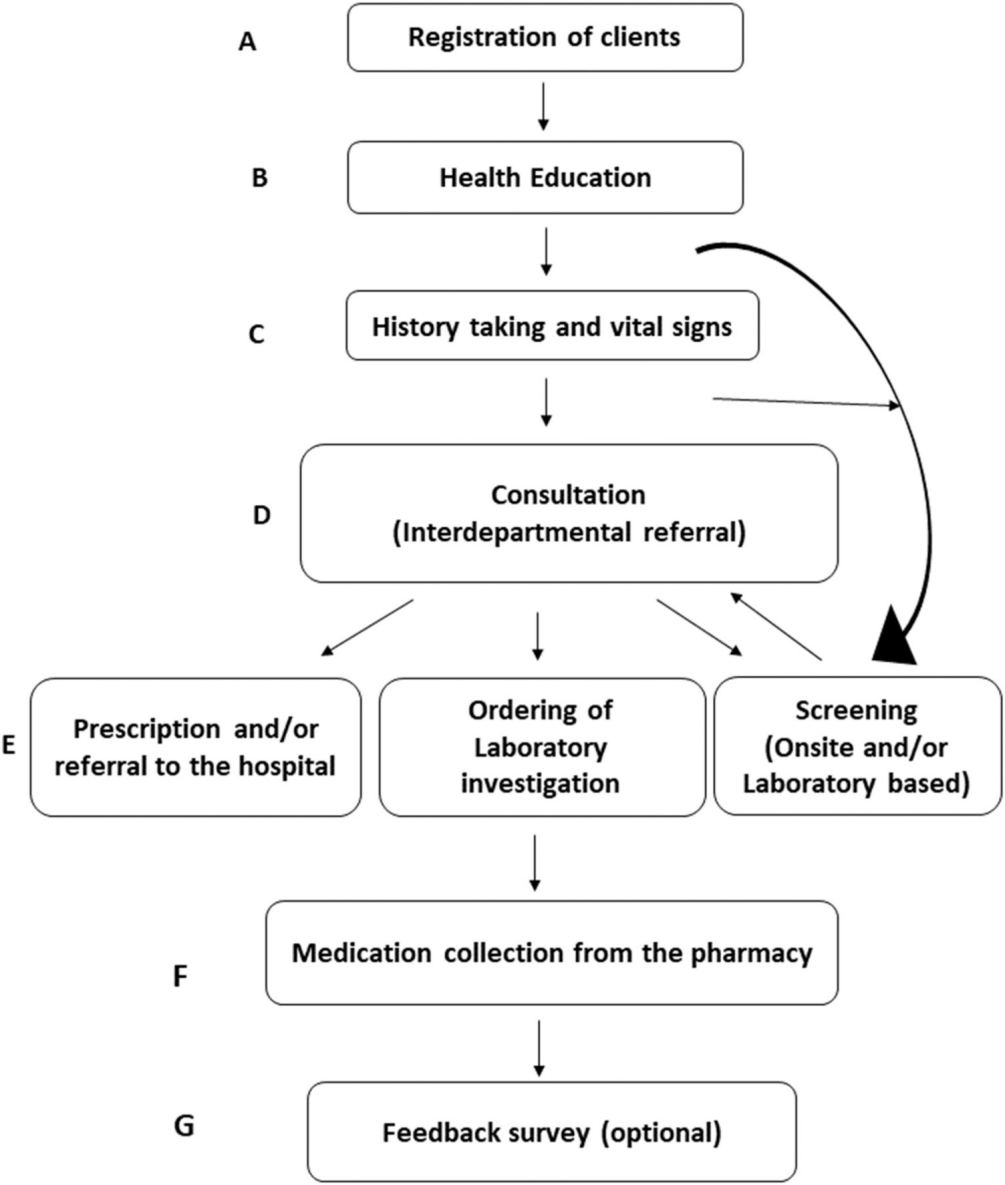
Flow diagram describing the Tabase community health outreach programme. **(A)** Registration of attendees; **(B)** delivery of health education; **(C)** history taking and vital signs; **(D)** disease screening pathways, including tuberculosis (TB), HIV, cervical cancer, prostate cancer and other services; **(D)** multidisciplinary consultation; **(E)** ordering of laboratory investigation, onsite and/or laboratory based screening; sometimes take the results from screening to consultation rroms for further discussion; **(F)** medication collection and **(G)** voluntarily provide feedback.

During the health outreach, the following activities were conducted and are presented in Figure 3.

– Registration of clients
– Health education on selected medical conditions at the reception area by different team members and postgraduate students
– History taking of the presenting health problem
– Taking of vital signs (sometimes key vital signs were taken before history taking) and screening
– Triage to one of the multidisciplinary teams for initial consultation
– Inter-departmental referral to a second professional team for a second opinion, further consultation, or in case of another unrelated complaint for a new consultation (clients had the unique discretion of consulting with various specialist teams in cases of comorbidities, such as hypertension, dental issues, or visual impairment. Conversely, immediate inter-specialty consultation and referrals were arranged according to the clients’ clinical features)
– Some clients were sent for HIV or TB screening and take the results to the doctor for further discussion
– Prescription and plan for follow-up at Tabase clinic or referral to hospital, ordering of laboratory investigation or screening
– Collection of medication from Tabase clinic pharmacy
– Feedback survey in which participation was voluntary

Tabase health outreach offered postgraduate students and researchers an opportunity to also conduct surveys on the knowledge, attitudes and practices of cervical cancer and tuberculosis. These surveys were independent of the Tabase health outreach we are currently reporting. It is important to mention that these surveys were approved by the WSU Health Research Ethics Committee and Eastern Cape Department of Health for degree and non-degree purposes. While MBChB-4 and 6 students were providing the service and health promotion, they also received education, similar to what is prescribed in the curriculum of MBChB community-based education and service. The Tabase health outreach programme functioned as a multi-component initiative integrating service delivery, education, and research. The report focuses on a retrospective audit and service evaluation of the outreach programme, focusing on service uptake, community feedback, and educational contributions.

## 4 Impact of Tabase health outreach

### 4.1 Tabase health outreach impact on service

**Figure 4:**
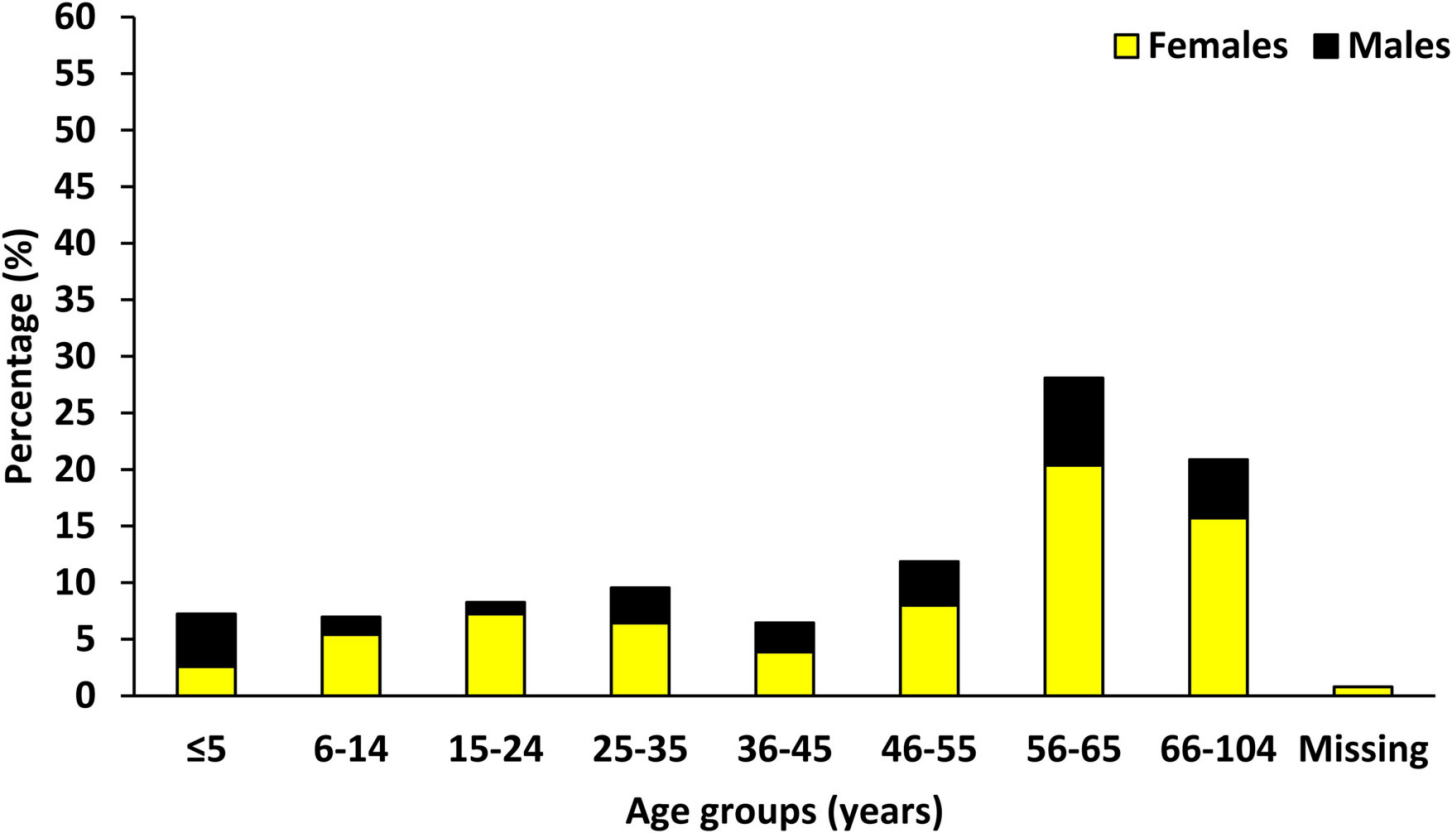
The distribution of Tabase health outreach attendees according to age and gender.

On the day of the Tabase Health Outreach programme, a total of 388 people were registered at the Tabase clinic; the majority were females (70.4%, 273/388), while males were the minority attendees (29.6%, 115/388). Age 56–65 years (28.1%, 109/388) was the major age group, followed by 66–104 years (20.9%, 81/388); while the least group was age group 6–14 years (7.0%, 27/388, Figure 4). In all age groups, women were the dominant attendees. According to Tabase clinic's daily statistics, they serve between 50 and 80 clients. This shows that on the day of health outreach, there was a 6-fold increase in the number of clients who attended the Tabase clinic for medical attention. Table 3 present the proportion of attendees according to villages and distance from the clinic. The Tabase health outreach program was attended by clients from villages as far as 41 km away from where the outreach was held.

**Table 3:** Distribution of Tabase health outreach attendees by the villages they reside in. .

Village name	*n*	%	km from Tabase clinic
Tabase	150	38.7	0–12.6^a^
Dumrana	53	13.7	5
Lugxogxo	28	7.2	9
Ngqunge	28	7.2	10
Tyeni	24	6.2	14
Mpheko plantation	19	4.9	10
Dukatole	17	4.4	20
Mdeni	10	2.6	1.4
Gubevu	9	2.3	13
Ntabeni	8	2.1	7
Mpafana	7	1.8	16.2
Mampingeni	6	1.5	11
Sixhotyeni	6	1.5	7
Manyosini	5	1.3	13
Cetulo (not catchment area)	4	1.0	13
Dlomo (not catchment area)	2	0.5	8
Mkwezweni	2	0.5	9
Langeni (not catchment area)	2	0.5	21.4
Lwandlana	2	0.5	16
Mbozisa	2	0.5	41
Xunu	2	0.5	16
Sigubudwini (not catchment area)	2	0.5	17.1

a Tabase is indicated as 0–12.6 km away from Tabase clinic because Tabase village has different subsections, in which the upper Tabase section is as far as approximately 12.6 km from Tabase clinic.

The clinical conditions commonly encountered at Tabase Clinic include hypertension, diabetes mellitus, epilepsy, pulmonary and extra-pulmonary tuberculosis (TB), asthma, schizophrenia, human immunodeficiency virus (HIV), various cancers, as well as the common cold and influenza. Community members were attended for some of these health conditions, and a few areas are discussed below. The TB/HIV Care team screened for TB, and after screening, 18 individuals qualified for TB laboratory testing; therefore, sputum samples were sent to the National Health Laboratory Service. All the results were negative for TB nucleic acid amplification tests. A total of 64 individuals were also screened for HIV infection, of which (53.1%, 34/64) were males and (46.9%, 30/64) were females. There were three newly diagnosed HIV cases (4.7%, 3/64), two were males (5.9%, 2/34), and one (3.2%, 1/31) was a female. The three newly diagnosed clients were initiated on antiretroviral therapy. An appointment to test the partner of one of the newly diagnosed clients was scheduled. The implementation of Men's Corner at the outreach contributed to the high level of men's participation in HIV testing. It is important to note that even though males represented approximately 30% of health outreach attendees, they represented 53% of those tested for HIV that day.

A total of 29 women received cervical cancer screening services, and 15 were seen by the gynecologist. The rehabilitation team successfully consulted 17 clients, with an age range of 14 to 95 years. After examination, a total of six individuals were provided with medical devices, including rollators and elbow crutches, to assist with walking. Tooth extractions were performed on 47 community members, of whom 76.6% (36/47) were female and 23.4% (11/47) were male. Children aged ≤12 years accounted for 27.7% (13/47) of the cases. Under normal circumstances, these patients would have been referred to Baziya Community Health Centre or Mthatha Regional Hospital, as Tabase Clinic does not provide dental services. There were few cases that were referred by the doctors to the hospital for further investigation and management.

### 4.2 Tabase health outreach impact on education and research

While community members were waiting at the reception area, health education on tuberculosis, human immunodeficiency virus, human papillomavirus and cervical cancer was conducted, and educational brochures were distributed. In certain cases, during consultation the individual education was also conducted. To promote sexually transmitted infection prevention, four boxes of male condoms were also distributed during the health education. The health outreach contributed to tuberculosis, human immunodeficiency virus, human papillomavirus and cervical cancer knowledge among the community members. Even though the impact of the delivered education was not measured, continuous health promotion is believed to contribute to improved knowledge and behavior (20).

The health outreach also contributed to the learning of WSU MBChB level four and six, BMed science honours and Masters’ students of WSU faculty of medicine and health sciences. Under the guidance of a qualified medical doctor, MBChB students contributed to taking vital signs and history, conducting physical examination, specimen collection, management, and consultations. While the Honours and Master's students as well as early carrier researchers contributed to the health education of community members. In addition, health outreach offered them an opportunity to conduct surveys on the knowledge, attitudes and practices of cervical cancer and tuberculosis. These surveys were approved by WSU Health Research Ethics Committee, and the collected data is of good quality and publishable. While students were offering the service and health promotion, they also received education, similar to what is prescribed in curriculum of community-based education and service.

## 5 Community feedback on Tabase health outreach

### 5.1 Community feedback survey introduction

A structured, voluntary feedback survey was conducted as part of the routine programme evaluation to assess community perceptions of the outreach. The survey instrument was adapted from established health service evaluation frameworks and community engagement assessment tools, incorporating domains commonly used in primary healthcare quality assessments, including service accessibility, perceived quality of care, organizational efficiency, relevance of services, and patient satisfaction. The questionnaire was contextually tailored to reflect the rural outreach setting and the multidisciplinary nature of the intervention; however, it was not validated prior to use. After attendees completed their clinical visit and/or collected their medication during the health outreach and exiting the health facility, they were requested to provide feedback on health outreach by completing a semi-structured questionnaire. The feedback provision by community members was voluntary. The structured questionnaire captured brief demographics, health outreach awareness channels, services used, perceptions of health outreach quality and organization, operational challenges, and intended follow-up. No personal identifiers were collected, and all responses were recorded anonymously. The questionnaire comprised both closed-ended and open-ended items. Perception-based items were measured using a five-point Likert scale (1 = strongly disagree to 5 = strongly agree). Open-ended questions were included to capture qualitative insights and participant suggestions for improvement (Supplementary Material S1).

#### 5.1.1 Statistical analysis of feedback survey

Data were coded, cleaned, and analysed using descriptive and inferential statistical methods. Categorical variables were summarized using frequencies and percentages, while continuous variables were summarized using measures of central tendency and dispersion (mean, median, and range). Prior to analysis, the internal consistency reliability of the Likert-scale items was assessed using Cronbach's alpha coefficient. The instrument demonstrated acceptable internal consistency (Cronbach's α ≥ 0.70), supporting the reliability of the composite perception domains. Likert-scale responses were analysed by calculating mean scores for each domain, and one-sample statistical tests were used to assess whether mean ratings differed significantly from a neutral midpoint. Associations between selected variables were explored using chi-square tests or appropriate non-parametric equivalents. In addition, an exploratory factor analysis (Omega analysis) was conducted to identify underlying latent constructs within the perception domains and to examine relationships between organizational, quality, and relational components of the outreach experience. Factor loadings and path coefficients were used to interpret clustering patterns and interrelationships among variables. This component of the study is reported in accordance with Strengthening the Reporting of Observational Studies in Epidemiology (STROBE) guidelines for cross-sectional studies (21), ensuring transparency in study design, data collection, analysis, and reporting.

Perception-based items measured on a five-point Likert scale were grouped into predefined domains, including satisfaction, institutional commitment, relevance, respect, awareness, perceived quality, organization, and usefulness of educational materials. For each domain, composite scores were calculated by averaging item responses, where applicable. Descriptive statistics were used to summarize responses, including mean scores and standard deviations (SD). To quantify the precision of the estimates, 95% confidence intervals (CI) for the mean were calculated using standard parametric methods. To assess whether community perceptions were significantly different from a neutral position, one-sample t-tests were conducted to compare each domain's mean with the neutral midpoint of the Likert scale (score = 3). This approach is commonly applied in Likert-scale analysis to evaluate whether observed perceptions are significantly positive or negative relative to neutrality. Statistical significance was set at *p* < 0.05. All tests were two-tailed. The magnitude and direction of differences were interpreted in relation to both statistical significance and practical relevance of mean scores. All analyses were conducted using R software, Version 4.3.1 (R Foundation for Statistical Computing, Vienna, Austria).

### 5.2 Results of community feedback survey

#### 5.2.1 Outreach awareness and services utilized

Out of 388 health outreach attendees, only 79 provided feedback during the Tabase Clinic Health Outreach. The age profile of respondents shows a median of 58.5 years, with participants ranging from 15 to 94 years old. The gender distribution was predominantly female (83.5%, 66/79), compared to 15.2% (12/79) male, with one respondent not disclosing age or gender.

Awareness about the health outreach to be held at Tabase clinic was driven mainly by the clinic staff and local networks, with all respondents citing “Other” sources and 7.6% through community leaders. This highlights the importance of formal clinic-community partnerships while leaving room to strengthen traditional leader and peer-mobilization pathways. Among those who provided feedback, the most accessed services were health screening (34.2%), immunization (20.3%), and dental services (16.5%), with fewer participants using health education (8.9%) and testing (8.9%). Although a chi-square test (*χ*^2^ = 0.00, *p* = 1.00) showed no statistically significant difference across categories, the descriptive results clearly indicate that screening and immunization were most in demand, reflecting community priorities. The observed high demand of dental services points to an unmet gap in routine care as it is not routinely offered at Tabase clinic.

#### 5.2.2 Perceptions of quality, relevance, and organization

Across eight domains, all ratings were significantly above neutral (*p* < 0.05), with means ranging from 4.08 to 4.84. The highest scores were for satisfaction (4.84), WSU commitment (4.75), and relevance (4.74); moderately high for welcomed & respected (4.68), awareness increased (4.62), and quality (4.63); and slightly lower, though still positive, for organization (4.35) and usefulness of talks/materials (4.08). All domains assessing perceptions of quality, relevance, and organization scored significantly above neutral (*p* < 0.05), confirming the strong acceptance of the health outreach program; strong trust and appreciation for respect and institutional presence, while pointing to opportunities to refine logistics and educational delivery. To provide a more detailed statistical summary, Table 4 presents domain-specific mean scores, standard deviations, 95% confidence intervals, and one-sample t-test results comparing each domain against the neutral midpoint (Likert score of 3). All domains remained significantly above neutral (*p* < 0.001), confirming strong positive community perceptions (Table 4).

**Table 4:** Community perception scores across outreach domains compared to neutral midpoint (Likert scale score of 3).

Domain item	Mean	SD	95% CI (lower–upper)	Test vs. neutral (3)	*p*-value
Satisfaction	4.84	0.42	4.75–4.93	t = 28.5	<0.001
WSU commitment	4.75	0.48	4.64–4.86	t = 26.1	<0.001
Relevance of Services	4.74	0.50	4.63–4.85	t = 25.7	<0.001
Welcomed & Respected	4.68	0.55	4.55–4.81	t = 23.4	<0.001
Awareness increased	4.62	0.60	4.48–4.76	t = 21.2	<0.001
Quality of services	4.63	0.58	4.50–4.76	t = 22.0	<0.001
Organization of services	4.35	0.72	4.18–4.52	t = 16.8	<0.001
Usefulness of talks/materials	4.08	0.80	3.89–4.27	t = 11.9	<0.001

Values represent mean Likert-scale ratings (1–5). One-sample t-tests were conducted comparing each domain mean against the neutral midpoint (Likert-scale score = 3).

The high perception scores across all eight domains indicate that community outreach efforts are both well-received and trusted by rural populations, which has clear implications for future initiatives. First, the exceptionally high satisfaction, institutional commitment, and relevance scores highlight the value of maintaining strong university visibility and sustained engagement in underserved areas, as this institutional trust can serve as a foundation for ongoing health promotion and research collaborations. Second, the slightly lower though still positive scores for organization and usefulness of materials point to actionable areas for quality improvement, such as enhancing logistics, communication flow, and tailoring educational resources to local literacy levels and cultural contexts. These refinements can increase efficiency and deepen learning impact during future events.

Moreover, the statistically significant positive ratings across all domains suggest that outreach programs do more than deliver episodic services they strengthen community confidence in formal healthcare systems and can act as gateways to continued care-seeking. Integrating feedback into planning can thus ensure iterative program design, where each outreach not only addresses immediate needs but also informs policy and training for community-based health models. Future efforts should institutionalize structured feedback mechanisms, include peer and traditional leader mobilization, and use these perception metrics to benchmark improvements in service delivery and community engagement over time.

#### 5.2.3 Operational challenges & intended actions

**Figure 5:**
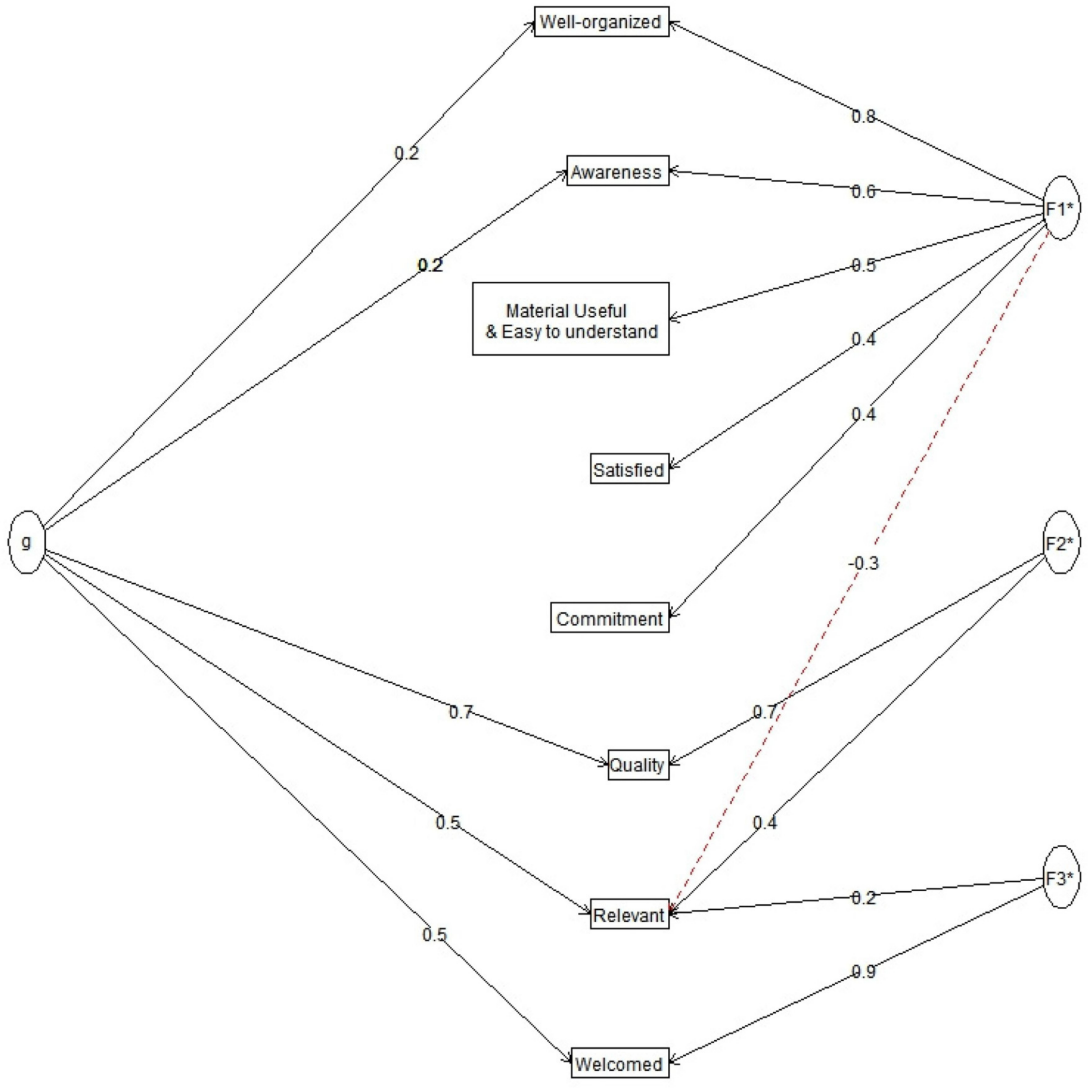
Omega plot showing how community members perceptions about the tabase health outreach (*n* = 79) are grouped into three themes: organisation, quality/relevance, and respect, with relevance bridging across them.

Three latent constructs emerged, representing clusters of related perceptions. Factor 1 (F1) captured domains related to organization and clarity, with strong loadings for *Well-organized* (0.8), *Awareness* (0.6), and *Material usefulness/ease of understanding* (0.5). Factor 2 (F2) reflected *Quality* (0.7) and *Relevance* (0.7), while Factor 3 (F3) was primarily driven by *Welcomed/respected* (0.9). The path coefficients highlight that *Commitment* and *Satisfaction* contributed moderately to F1 (0.4 each), reinforcing the connection between organizational aspects and participant satisfaction. A notable negative correlation (–0.3) was observed between F1 and F2, suggesting that perceptions of logistical organization were inversely related to perceived quality and relevance. By contrast, *Relevance* maintained a cross-loading with both F2 (0.7) and F3 (0.4), underscoring its bridging role between service quality and relational aspects of respect (Figure 5).

About a quarter of respondents cited long waiting times, flow, or accessibility issues, signaling the need for improved queue management, signage, and staff distribution. Importantly, 46.8% reported intentions to take action towards their health, such as follow-up visits or lifestyle changes, showing the outreach not only delivered immediate services but also stimulated continued health engagement or seeking behaviour. Even though these may be short-term, continuous engagement may enhance them.

#### 5.2.4 Community suggestions for improvement

Community members called for more staff to reduce waiting times, better access to medication, expanded dental services, and improved logistics (directions, seating, triage). Among the services not provided during the health outreach, community members requested an ophthalmologist and an ear, nose and throat specialist. These suggestions demonstrate high trust but also high expectations, with communities seeking comprehensive, accessible, and efficiently organized services.

## 6 Discussion

This report demonstrates the contribution of community engaged health outreach program towards health service delivery, community education, health science student education and research in rural Eastern Cape Province of South Africa. Women were the major attendees of the Tabase health outreach. This pattern aligns with broader trends in community health programmes, where women are more likely to attend health services and act as health information gatekeepers (22, 23). This underscores the opportunity to leverage women's participation for family health impact, while also highlighting the need to encourage greater male attendance, particularly for STIs, TB/HIV screening. However, it was interesting to note high male participation in HIV testing during the outreach, and this was attributed by having a dedicated Men's Corner, which created a targeted and supportive environment for male attendees. Evidence consistently shows that men underutilize HIV testing and other health services due to structural and cultural barriers, including perceptions that clinics are female-oriented spaces (19, 24). By adapting a men-friendly clinic strategy, the outreach addressed these barriers, normalizing health-seeking behavior and encouraging greater male engagement. Similar initiatives, such as male-friendly spaces and differentiated service delivery models, have been shown to improve men's uptake of HIV testing and treatment services in sub-Saharan Africa (25, 26). These findings highlight the importance of gender-sensitive approaches that reframe service delivery environments to address men's specific health needs while sustaining broader community participation. It is recommended that health facilities without men-friendly strategies must initiate similar strategies to improve men's health in their communities.

The Tabase health outreach program was attended by clients from villages as far as 41 km away from where the outreach was held. Some of these villages are also part of the normal Tabase clinic catchment area. The distance between the dwelling villages of the attendees and the nearest primary healthcare facility exceeds the recommended 5 km distance (4). The long distance between the community members' residences and the nearest health facilities has been reported in Eastern Cape Province (4). This means that travelling to the nearest health facilities involves direct costs (transport) and indirect costs (time), leading to delayed care, fewer routine visits, and reliance on outreach events. Health-seeking behavior could therefore be constrained not only by awareness but also by the financial and logistical feasibility of reaching care (27, 28). Evidence from rural South Africa and other sub-Saharan settings shows that long travel distances contribute to missed appointments, poor treatment adherence, and higher attrition in TB/HIV programmes (29, 30). These indicate how distance to the nearest health facility strongly shapes healthcare utilization. The Tabase outreach reduced these barriers by bringing services that are not usually found in the Tabase clinic, but at the referral hospital and community health center, resulting in a six-fold increase in attendance. These findings point to the need for strategies that reduce the cost–distance burden, including mobile clinics, decentralized drug distribution, and community health worker follow-up. Transport support or coordinated community transport could further improve equity and sustain health-seeking behavior across all catchment areas.

The community members' feedback about the health outreach indicated the highest ratings for satisfaction, institutional commitment, and relevance, underscoring the importance of health outreach initiatives that engage the community (31, 32). Moderately high scores for respect, awareness, and quality reinforce evidence that participatory approaches foster inclusivity and facilitate knowledge translation in resource-constrained settings (33). Although organization and usefulness of materials received slightly lower ratings, these remain positive, suggesting logistical and pedagogical refinements could further enhance effectiveness, consistent with findings that culturally appropriate, interactive formats improve community health education (34). The findings indicate an overwhelmingly positive reception across all eight domains assessed, with all mean scores significantly above the neutral midpoint. This demonstrates that participants not only engaged meaningfully with the initiative but also valued its overall quality and impact and strong trust in Walter Sisulu University Faculty of Medicine and Health Sciences and other health partners.

Long queues and inefficient patient flow are well-documented barriers to care in resource-limited settings, contributing to patient dissatisfaction and delayed health-seeking behavior (35, 36). The concerns raised in this study highlight the need for practical interventions, including improved queue management systems, clearer signage to guide movement, and more strategic staff distribution to balance workloads. Similar recommendations have been shown to reduce waiting times and enhance patient experiences in primary healthcare clinics (37, 38). Addressing these operational issues is therefore not only likely to improve efficiency but also to strengthen trust and uptake of services, particularly in high-burden rural contexts. Awareness about the health outreach to be held at Tabase clinic was driven mainly by the clinic staff and local networks, with a few reported through community leaders. This highlights the importance of formal clinic-community partnerships while leaving room to strengthen traditional leader and peer-mobilization pathways.

The Omega analysis underscores that community perceptions of outreach services are not unidimensional but shaped by interlinked constructs of organization, relevance, and respect. The strong clustering of “well-organized”, “awareness”, and “usefulness of materials” suggests that logistical clarity and communication are central to perceived program effectiveness, aligning with evidence that health interventions must be both well-structured and easily understood to achieve meaningful uptake (39, 40). At the same time, the bridging role of relevance across factors indicates that participants judge services not only on delivery logistics but also on whether they address locally felt needs a finding consistent with research on community-engaged models of care, where contextual tailoring drives trust and sustained participation (31, 32). The high loading of welcomed/respected highlights the relational dimension of service delivery, echoing studies that patient respect and dignity are critical determinants of satisfaction and future health-seeking behavior (41, 42). Importantly, the observed negative association between organizational perceptions and quality/relevance may reflect heightened expectations: when logistical shortcomings occur, they disproportionately shape judgments of overall quality. These insights reinforce the importance of integrating operational efficiency with participatory, respectful engagement in order to maximize both trust and impact of community-based outreach. It is important to indicate that the community perception analysis was limited by a small sample of community members who provided feedback (*n* = 79). We acknowledge the gendered utilisation patterns (83.5% female), the sample size for males who responded to the feedback was 12, and too small to provide statistically robust separate factor structures. These perceptions might differ across genders in larger cohorts; therefore, this data must be used with caution.

### 6.1 Lessons learned and recommendations

A reflection session was conducted a week later among the team members (Walter Sisulu University, Eastern Cape Department of Health, National Health Laboratory Service and Tabase clinic committee). The lessons learned and recommendations by the team members are presented below and none of the recommendations were already implemented.

– Proper preparation is required when planning the community health outreach. Understanding the environment in which the health outreach will be conducted is important for successful outreach. Even though the team did prepare for the outreach, having a mock visit, map-up the facility, briefing sessions on how the health outreach team will work were necessary steps to be part of preparation. This was going to contribute in standardizing certain procedures between consultation.
– Understanding the community is essential for accurate resource planning and preventing critical supply shortages, as demonstrated during this outreach, when the dentist had to stop treating patients because the anesthesia ran out.
– Separate clients who have an appointment for that day or who come to collect their medication; have a dedicated area for those booked for the day and those attending an outreach. However, those who were booked for the day may also participate and benefit from the outreach services if they desire.
– Since the clients arrive hours before the normal clinic opening time, they can be registered earlier than the normal clinic hours. For example, the client's registration and folder retrieval start early, as do vital signs and history taking. This will assist in sorting the clients and direct them to the relevant consultation room, so that the doctors can start immediately upon arrival. This could significantly improve the workflow.
– After considering the catchment area statistics, getting more spacious facilities and/or adding more mobile clinics/ tents to the consultation rooms could be beneficial. At Tabase health outreach, the number of health professionals exceeded the available consultation rooms; as a result, some consultation rooms had more than one doctor.
– Add more vital sign taking machines. This can be done by not only purchasing new ones but by borrowing from a nearby health facility.
– Improve transport logistics for health professionals to attend the outreach.
– Providing transport to clients from far could increase the attendance of those in need, but with no transport fee.
– Making sure that at the outreach site, the basic needs are available, like running clean water, toilets, and chairs to sit.

## 7 Conclusion

The community-engaged outreach programme demonstrated potential to contribute to health service offerings, health education for community members, and undergraduate and postgraduate students’ learning and community-based research. Community members' perceptions of community health outreach were overwhelmingly positive, with mean ratings being highest for satisfaction, WSU commitment, and relevance. Unfortunately, community members faced challenges during the health outreach programme. However, while almost half planned follow-up action, showing that the outreach served as both a service point and a catalyst for continued health-seeking behavior. The Tabase outreach was both statistically and practically significant in meeting community needs, especially in building trust and delivering preventive services.

## Data Availability

The raw data supporting the conclusions of this article will be made available by the authors, without undue reservation.
