# Supplementary material for: Community-engaged health outreach program contributes to health service, research, and education in rural areas
Source: Front Health Serv. 2026 May 12;6:1749125. doi: 10.3389/frhs.2026.1749125 (PMC13201518; doi:10.3389/frhs.2026.1749125)
Supplement: Supplementary file 1 [file Datasheet1.pdf]

Community feedback questionnaire

Tabase Clinic Health Outreach

Date: 28 August 2025

We value your feedback to help us improve future health outreach programs. Please take a few minutes to complete this questionnaire.

Section A: General information

Age: ____________________

Gender: ____________________

Section B: Participation and experience

Please tick (✓) your answer.
(1 = Very Poor, 2 = Poor, 3 = Fair, 4 = Good, 5 = Excellent)

1. How did you hear about this health outreach program?

☐ Community leader ☐ Word of mouth ☐ Other: ____________

2. Which services did you use during the outreach? (tick all that apply)

☐ Health screening ☐ Health education ☐ Immunization ☐ Dental ☐ Testing (e.g., TB, HIV, BP, diabetes) ☐ Counseling ☐ Other: ____________

3. How satisfied were you with the health services provided today?

☐ 1 ☐ 2 ☐ 3 ☐ 4 ☐ 5

4. Did you feel welcomed and respected by the staff and health professionals?

☐ 1 ☐ 2 ☐ 3 ☐ 4 ☐ 5

5. Were the services (e.g., TB/HIV, cancer screening, dental, physiotherapy) relevant to your community's needs?

☐ 1 ☐ 2 ☐ 3 ☐ 4 ☐ 5

6. How would you rate the quality of the screenings and consultations you received?

☐ 1 ☐ 2 ☐ 3 ☐ 4 ☐ 5

7. Were the health talks, engagement sessions, and educational materials useful and easy to understand?

☐ 1 ☐ 2 ☐ 3 ☐ 4 ☐ 5

8. Was the outreach well organized in terms of venue, time, and access to services?

☐ 1 ☐ 2 ☐ 3 ☐ 4 ☐ 5

9. Did you face any challenges (e.g., long waiting times, unclear directions)?

☐ Yes ☐ No

If yes, please explain: ____

____

____

Section C: Community impact

10. Do you feel this outreach increased your awareness of important health issues?

☐ 1 ☐ 2 ☐ 3 ☐ 4 ☐ 5

11. How well do you think Walter Sisulu University showed its commitment to serving your community?

☐ 1 ☐ 2 ☐ 3 ☐ 4 ☐ 5

12. Do you plan to take any action after this outreach (e.g., follow-up clinic visit, lifestyle changes, sharing knowledge)?

☐ Yes ☐ No If yes, please explain: ___________________________

Section D: Suggestions for the future

13. What additional services, topics, or improvements would you like to see in future health outreach activities?

____

____

____

Thank you for your time and feedback!

Your responses will help us improve our faculty's community engagement and health outreach activities.

Regards

Prof ZZA Mbulawa
